# Acute Appendicitis or Appendiceal Diverticulitis? A Case Report and Systematic Literature Review

**DOI:** 10.3390/clinpract15030060

**Published:** 2025-03-13

**Authors:** Stipe Vidović, Nenad Čekić, Ivica Šuvak, Mladen Ugljarević, Zenon Pogorelić

**Affiliations:** 1 Department of Surgery, National Memorial Hospital Vukovar, 32 000 Vukovar, Croatia; 2 Faculty of Medicine, Josip Juraj Strossmayer University of Osijek, 31 000 Osijek, Croatia; 3 Faculty of Dental Medicine and Health, Josip Juraj Strossmayer University of Osijek, 31 000 Osijek, Croatia; 4 Department of Surgery, School of Medicine, University of Split, 21 000 Split, Croatia; 5 Department of Pediatric Surgery, University Hospital of Split, 21 000 Split, Croatia

**Keywords:** appendix, acute appendicitis, appendectomy, appendicitis, appendiceal diverticulitis, appendiceal diverticulosis, diverticulitis

## Abstract

**Background**: Appendiceal diverticulitis is a rare and poorly understood condition of the appendix. The diagnosis of appendiceal diverticulitis is challenging due to its rarity and a clinical presentation that often mimics other ileocecal disorders. Unlike acute appendicitis, appendiceal diverticulitis may be associated with a higher risk of perforation, increased mortality, and a potential link to neoplasms. However, further research is necessary to enhance our understanding of its epidemiology, risk factors, clinical presentation, and outcomes. **Case Report**: A 53-year-old male presented to the emergency department with right lower abdominal pain. On physical examination, tenderness was noted in the right lower quadrant, without rebound tenderness or muscle guarding. Laboratory tests revealed leukocytosis and elevated C-reactive protein (CRP) levels. Ultrasonographic imaging of the ileocecal region suggested acute appendicitis, leading to a decision for surgical intervention. Laparoscopic exploration revealed multiple cylindrical, red, and edematous herniations, up to 4 mm in size, on the surface of the vermiform appendix. An appendectomy was performed. Histopathological examination confirmed appendiceal diverticulitis with surrounding peridiverticulitis. The surgery and early postoperative course were uneventful. **Literature review**: The study included 5 retrospective studies and 30 case reports, analyzing a total of 112 patients with appendiceal diverticulitis. Of these, 65.5% were male and 34.5% were female, with a median age of 49 years (IQR: 39–59). The most commonly reported clinical findings included pain in the right iliac fossa or right lower abdominal quadrant (56.5%), nausea (18.9%), vomiting (9.8%), rebound tenderness (24.6%), fever (15.6%), leukocytosis (25.4%), and elevated C-reactive protein levels (16.4%). Diagnosis was confirmed histopathologically in 86.9% of the cases via computed tomography imaging in 4.1% and ultrasonography in 1.6%. A histopathological analysis identified five neoplasms (4.1%), including two sessile serrated adenomas, two neuroendocrine carcinoids, and one mucinous tumor. Appendectomy was the treatment of choice, with no intraoperative or postoperative complications recorded and no mortality reported. The median hospital stay was 6.8 days (IQR: 3.0–6.8). **Conclusions**: Appendiceal diverticulitis should be considered as a differential diagnosis in patients presenting with symptoms resembling acute appendicitis. Early diagnosis and treatment are essential to reduce morbidity and mortality. Appendectomy is a safe and effective treatment approach for appendiceal diverticulitis.

## 1. Introduction

The vermiform appendix, a small tubular structure attached to the cecum in the lower right quadrant of the abdomen, has been a subject of anatomical, physiological, and pathophysiological interest for centuries [1]. Among the spectrum of appendiceal pathologies, acute appendicitis stands out as the condition with the highest incidence, representing one of the most common surgical emergencies [2,3]. The clinical presentation of acute appendicitis typically includes right lower quadrant abdominal pain, fever, and leukocytosis [4]. However, the diagnosis is not always straightforward due to the variability in clinical presentation and the broad spectrum of potential differential diagnoses of acute appendicitis, which underscores the clinical challenges in accurately diagnosing appendiceal disorders [5,6].

One of the clinical entities that may mimic acute appendicitis is appendiceal diverticulitis. Studies estimate the prevalence of appendiceal diverticulosis in appendectomy specimens to range from 0.004% to 2.1% [7]. Despite being first described by the pathologist T.H. Kelynack in 1893, appendiceal diverticulitis remains a poorly understood and infrequently studied entity [8].

In this article, we present a case report of a 53-year-old male patient diagnosed with appendiceal diverticulitis whose clinical presentation resembled that of acute appendicitis. Furthermore, a review of the literature describing appendiceal diverticulitis is provided.

## 2. Methods

A literature search (by S.V.) on appendiceal diverticulitis was conducted on 10 December 2024 using the following four electronic databases: PubMed, Scopus, Web of Science, and ScienceDirect. Boolean logical operator expressions were used to search within the databases, as follows:

PubMED: (“appendice”[All Fields] OR “appendiceal”[All Fields] OR “appendices”[All Fields]) AND (“diverticulitis”[MeSH Terms] OR “diverticulitis”[All Fields]) AND (“appendix”[MeSH Terms] OR “appendix”[All Fields] OR “appendix s”[All Fields] OR “appendixes”[All Fields]) AND (“diverticulitis”[MeSH Terms] OR “diverticulitis”[All Fields]).

Scopus: ((appendiceal AND diverticulitis) AND (appendix)) AND (diverticulitis). AND (LIMIT-TO (SUBJAREA, “MEDI”)) AND (LIMIT-TO (DOCTYPE, “ar”)) AND (LIMIT-TO (EXACTKEYWORD, “Human”)) AND (LIMIT-TO (LANGUAGE, “English”)) AND (LIMIT-TO (SRCTYPE, “j”)).

Web of Science: TS = ((appendiceal diverticulitis) AND (appendix)) AND (diverticulitis)).

ScienceDirect: ((appendiceal diverticulitis) AND (appendix)) AND (diverticulitis). The search was restricted to papers in English and with the access type Open Access and Open Archive.

The inclusion and exclusion criteria for the selection of the studies are noted in Table 1.

**Figure 1:**
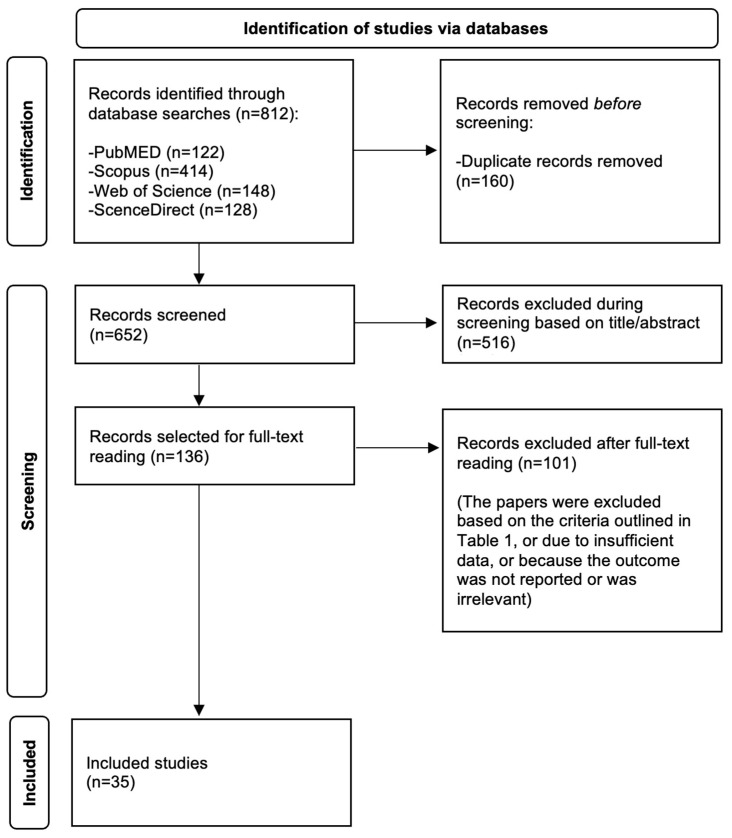
A flow diagram of the literature search.

A search of the databases identified 812 records. After removing 160 duplicates before the screening phase, 652 records remained. During screening, 516 records were excluded based on titles and abstracts. Subsequently, 136 full-text papers were assessed, of which 101 were excluded based on the inclusion and exclusion criteria (Table 1) due to insufficient data or because the reported outcome was either missing or irrelevant. Ultimately, 35 studies were included in the study. A flow diagram of the literature search is presented in Figure 1.

The data extraction, performed by S.V. and Z.P., focused on studies of appendiceal diverticulitis and included the following variables: the first author of the article, year of publication, study design, total number of participants, gender distribution, study period, reported symptoms, findings from physical examinations and laboratory tests, diagnostic methods, associated neoplasms, management strategies, intraoperative and postoperative complications, length of hospital stay, and mortality rates.

## 3. Case Report

A 53-year-old male patient presented to an emergency department with pain in the right hemiabdomen. The pain had begun earlier that morning, localized in the right lower quadrant of the abdomen, and it progressively intensified throughout the day. That morning, the patient experienced mild nausea without vomiting. Prior to this, the patient had not experienced any similar episodes or pain in this region. Furthermore, the patient reported no history of chronic illnesses or prior surgeries.

Upon admission, the patient was afebrile, and his vital signs were normal. Abdominal palpation revealed pain (8/10) without guarding, and it was most intense in the right lower quadrant of abdomen. The Blumberg sign, Rovsing sign, and obturator sign were negative. Hematological tests revealed leukocytosis (20.9 × 10^9^/L), while biochemical tests showed elevated C-reactive protein (CRP) levels (22.4 mg/L). An ultrasound of the abdomen revealed a tubular, non-compressible structure with dorsal enhancement adjacent to the ileocecal region at the cecal base, measuring up to 25 mm in diameter, surrounded by mesenteric fat. There was no evidence of free fluid, diffuse peritonitis, or appendicolith.

**Figure 2:**
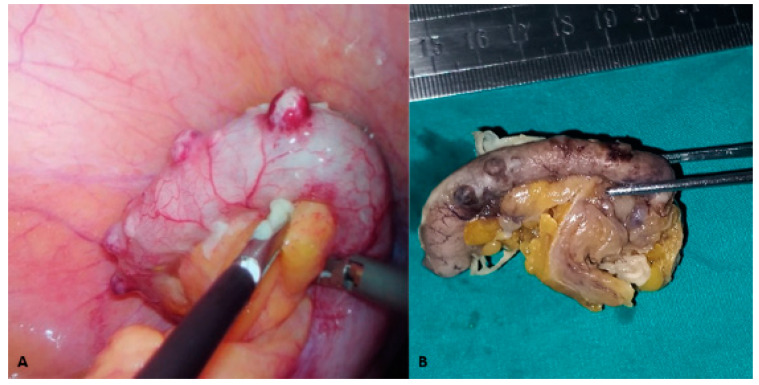
Macroscopic visualizations of the vermiform appendix: (**A**) the intraoperative findings for the vermiform appendix, with multiple cylindrical, red, and edematous herniations on its surface; and (**B**) the macroscopic appearance after embedding the appendix in 4% buffered formalin, with several visible focal protrusions measuring up to 0.4 cm in diameter.

The Appendicitis Inflammatory Response (AIR) score was 6, indicating a mild probability of acute appendicitis [9]. Based on the patient’s overall symptoms, physical examination findings, laboratory results, and ultrasound findings, laparoscopic exploration was indicated and carried out without further diagnostic evaluation.The laparoscopic exploration revealed cylindrical, red, and edematous herniations on the surface of the appendix (Figure 2A). An appendectomy was performed, and the appendix was sent for histopathological examination. Regarding histopathological findings, the appendix measured 6 cm in length macroscopically, with surrounding fatty tissue, having a diameter of 5.5 cm. The wall exhibited focal protrusions, up to 0.4 cm in diameter, located on the mesenteric edge of the appendix (Figure 2B).

**Figure 3:**
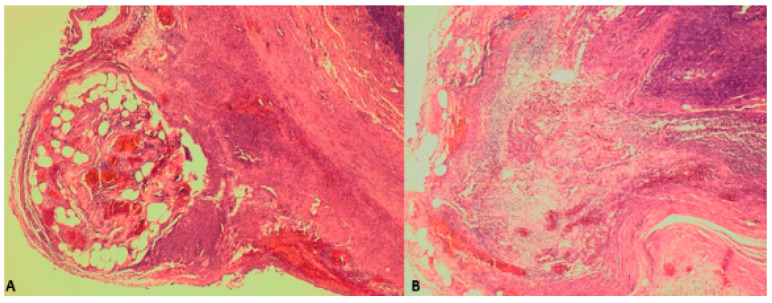
The histopathological findings: (**A**) herniation of the mucosal and submucosal layers through a defect in the muscular layer of the appendix; and (**B**) localized lymphoid follicular hyperplasia and an abundance of granulocytes and mononuclear cells, collectively indicating appendiceal diverticulitis.

Microscopically, herniation of the mucosal and submucosal layers through a defect in the muscular layer was observed. Additionally, lymphoid follicular hyperplasia, along with an abundance of granulocytes and mononuclear cells, was identified within the wall, extending into the described diverticular protrusions (Figure 3). Overall, these findings were indicative of appendiceal diverticulitis.

The surgery and early postoperative course proceeded without complications. During his hospital stay, the patient was treated with crystalloid infusions, antiemetics, and analgesics. He recovered well, tolerated oral feeding, and he had normal bowel movements. On postoperative day two, the patient was discharged home in good general and local condition. In the follow-up period of three months, the patient remained in good overall and local condition.

## 4. Discussion

Diverticulitis is most commonly described in the colon and, very rarely, in the appendix, as in our case [10]. Appendiceal diverticulitis can be classified as either congenital or acquired. In the congenital form, the mucosa, submucosa, and muscular layer herniate through the wall, whereas in the acquired form, only the mucosa and submucosa are involved [8]. Additionally, in the acquired form, herniations are typically more numerous, smaller (2–5 mm), and located on the mesenteric edge, whereas the congenital form is usually solitary, larger, and found on the antimesenteric edge of the appendix [11,12,13,14]. In our case, macroscopically, the diverticula measured up to 4 mm and were located on the mesenteric edge of the appendix. A histopathological examination revealed herniation of the mucosal and submucosal layers through a defect in the muscular layer, suggesting that our specimens were acquired diverticula. The acquired type is more common than the congenital type, which has an incidence of 0.014%, constituting approximately 3% of all appendiceal diverticula [7].

The classification of appendiceal diverticular disease was introduced by Phillips et al., who defined five distinct microscopic types of appendiceal diverticulitis (Table 2) [15]. Histopathologically, our specimen met the criteria for type one.

### 4.1. Review of the Literature

Following a review of the literature, the study included and analyzed 5 retrospective studies and 30 case reports, encompassing a total of 112 patients with appendiceal diverticulitis. Of these, 65.5% were male and 34.5% were female, with a median age of 49 years (IQR: 39–59). The most commonly reported symptoms were pain in the right iliac fossa or right lower abdominal quadrant (56.5%), nausea (18.9%), and vomiting (9.8%). Additionally, the most frequently observed physical examination and laboratory findings included rebound tenderness (24.6%), fever (15.6%), leukocytosis (25.4%), and elevated CRP levels (16.4%). The diagnosis was confirmed histopathologically in 86.9% of cases via CT imaging in 4.1% and through ultrasonography in 1.6%. A histopathological analysis identified five neoplasms (4.1%), including two sessile serrated adenomas, two neuroendocrine carcinoids, and one mucinous tumor. Table 3 presents the main characteristics and clinical findings of the patients with appendiceal diverticulitis.

The risk factors for appendiceal diverticulitis include chronic appendicitis, Hirschsprung’s disease, cystic fibrosis, an age of over 30 years, and being of male sex. In our case, the patient was older than 30 and male. Findings from case reports and retrospective studies also indicated that patients predominantly tended to be over 30 years old (Table 4). Additionally, a higher prevalence of appendiceal diverticulitis among men was reported in retrospective studies by Ergenç and Uprak, Yardimci et al., and Yamana et al. [20,36,40]. Moreover, Philips et al. provided a tabular overview of the symptomatology of appendiceal diverticulitis (Table 4), which largely aligned with the clinical presentations described in case reports presented in Table 2 [15]. Our patient also exhibited leukocytosis and elevated CRP levels, findings that were consistent with a majority of the case reports and retrospective studies on appendiceal diverticulitis (Table 3).

Studies have reported a high prevalence of perforation in appendiceal diverticulitis, ranging from 30% to 70%, which is four times higher than in appendicitis [15,20,36,38,45]. This increased incidence of perforation is primarily attributed to the thin-walled diverticulum, which serves as a weak point prone to rupture. Consequently, patients with appendiceal diverticulitis face a 30-fold higher mortality risk compared to those with simple appendicitis [15,45]. In our patient, no perforation was observed.

In the context of preoperative diagnosis, studies have indicated that ultrasound and CT imaging can be useful in identifying appendiceal diverticulitis, with a diagnostic accuracy of 86% in pathologically confirmed cases [46,47,48]. Furthermore, studies suggest that, when interpreted by experienced radiologists, most cases of appendiceal diverticulitis can be differentiated from acute appendicitis using CT imaging. This differentiation is based on the characteristic appearance of inflamed diverticula, which present as small cystic protrusions within the appendix, accompanied by an increased contrast enhancement of the diverticular wall [46,47,49]. However, other research has highlighted that these radiological techniques lack specificity for this condition [49,50,51].

Several studies have described an association between the presence of appendiceal diverticulosis and neoplasms such as carcinoid tumors and mucinous adenomas [49,50,51,52,53,54]. In our case, no neoplasm was identified.

A review of the literature and an analysis of the included studies established that appendectomies were performed in 98 patients with appendiceal diverticulitis. Among these, 20 laparoscopic and 14 open appendectomies were performed, while the type of appendectomy was unspecified in the remaining cases. Furthermore, no intraoperative or postoperative complications were recorded, and no mortality was reported. The median hospital stay was 6.8 days (IQR: 3.0–6.8) (Table 5). Notably, the studies indicated that prophylactic appendectomy was recommended for all patients in whom appendiceal diverticula were identified as an intraoperative finding due to the increased risk of perforation, malignancy, and associated mortality [12,52,53].

### 4.2. Comparison Between the Clinical and Laboratory Findings of Acute Appendicitis and Appendiceal Diverticulitis

Appendiceal diverticulitis should be considered in the differential diagnosis of patients presenting with right lower quadrant pain suggestive of acute appendicitis. The importance of this distinction lies in its clinical implications. Studies report a perforation risk of up to 70% in appendiceal diverticulitis compared to approximately 10–20% in acute appendicitis, leading to a significantly increased risk of sepsis and mortality [15,20,36,38,45]. Additionally, there is a well-documented association between appendiceal diverticulosis and neoplasms, particularly mucinous neoplasms, and carcinoid tumors, which may necessitate further surgery or postoperative surveillance [49,50,51,52,53,54,55,56,57,58]. Although imaging findings in appendiceal diverticulitis can be subtle, preoperative detection may require the use of contrast-enhanced computed tomography, which has demonstrated up to 86% accuracy in distinguishing this condition from acute appendicitis [46,47,48]. Given these considerations, identifying appendiceal diverticulitis preoperatively could lead to earlier surgical intervention, a reduced risk of complications, and more targeted postoperative follow-up, particularly in cases where histopathological findings indicate neoplastic changes.

Although appendiceal diverticulitis and acute appendicitis share similar clinical presentations, retrospective studies suggest subtle differences that may aid in diagnosis. Unlike acute appendicitis, appendiceal diverticulitis often presents with insidious pain that can persist for 2 to 14 days before hospitalization [15]. Furthermore, appendiceal diverticulitis is more frequently observed in patients over 30 years of age, whereas acute appendicitis is typically diagnosed in younger individuals [15,36,38]. Laboratory findings, such as leukocytosis and elevated CRP levels, occur in both conditions; however, appendiceal diverticulitis is more commonly associated with leukocytosis (>15 × 10^9^/L) and significantly higher CRP levels [38,45]. Moreover, appendiceal diverticulitis is four times more likely to result in perforation, further increasing the risk of postoperative complications and mortality [15,38,45]. Table 6 summarizes the key distinguishing features between these two conditions.

### 4.3. Limitations

This systematic review included 35 studies, of which 6 were retrospective studies and 29 were case reports, and all were single-centered and had relatively small sample sizes. Additionally, no retrospective cohort studies, prospective studies, or randomized controlled trials on appendiceal diverticulitis were identified. Moreover, numerous studies did not report or sufficiently describe the variables of interest, further limiting the comprehensiveness of the analysis, increasing the potential for bias, and restricting the generalizability of the findings. Due to the small number of studies and limited sample sizes, a meta-analytic approach to data synthesis was not undertaken. This further constrained the possibility of quantitative synthesis, increased subjectivity, made it more challenging to identify patterns, and prevented the assessment of heterogeneity.

Further retrospective cohort studies, prospective studies, and randomized controlled trials, preferably multi-centered and with a larger sample size, are needed to provide more comprehensive and unbiased evidence on appendiceal diverticulitis. Standardizing methodologies and reporting key variables across studies would enable more thorough analyses and facilitate meta-analyses. Such research could enhance the understanding of this still insufficiently characterized pathology.

## 5. Conclusions

This case report highlights the incidental discovery of appendiceal diverticulitis, which clinically mimicked acute appendicitis and was successfully treated with laparoscopic appendectomy. We emphasize the importance of considering appendiceal diverticulitis as a differential diagnosis in patients presenting with symptoms suggestive of acute appendicitis. Moreover, additional research is needed to better understand the risk factors, clinical manifestations, disease progression, and optimal treatment strategies for this condition.

## Figures and Tables

**Table 1:** Inclusion and exclusion criteria for the study.

	Inclusion Criteria	Exclusion Criteria
**Study design**	Case report and retrospective study (case series or case-control study)	Conference abstracts, editorials, commentaries, and personal communications
**Study period**	All available literature to date	/
**Language**	English	Languages that are not English
**Population**	Human participants of all age groups	Animal studies
**Study topic**	Appendiceal diverticulitis with/without the clinical presentation of acute appendicitis	Topics that are not associated with appendiceal diverticulitis with/without the clinical presentation of acute appendicitis

**Table 2:** Types of appendiceal diverticular disease according to classification by Phillips et al. [15].

Type 1	Primary acute diverticulitis, with or without acute peridiverticulitis
Type 2	Acute diverticulitis secondary to acute appendicitis
Type 3	Diverticulum without inflammation
Type 4	Diverticulum with acute appendicitis
Type 5	Chronic peridiverticulitis with acute appendicitis

**Table 3:** Main characteristics and clinical findings of the patients with appendiceal diverticulitis.

Author (Study Period, Country), Study Design	Sample Size (Male/Female)	Age	Symptoms	Physical Examination and Laboratory Findings	Method of Diagnosis	Associated Neoplasms
Abdelrahim et al. (2024, United Kingdom), case report [16]	1 (1/0)	50	Right iliac fossa abdominal pain	NA	Computer tomography (CT)	No neoplasm
Cadena et al. (2023, Colombia), case report [17]	1 (1/0)	42	3-day history of right inferior quadrant pain, fever, and hyporexia	A white-blood-cell count of 13.6 × 10^9^/L	Histopathology	No neoplasm
Laamiri et al. (2023, Tunisia), case report [18]	1 (1/0)	59	1-day history of right iliac fossa pain, nausea, and vomiting	A mean white-blood-cell count of 13 × 10^9^/L and elevated CRP levels (CRP = 55 mg/dL)	Histopathology	No neoplasm
Bonomo et al. (2022, Italy), case report [19]	1 (1/0)	22	1-day history of right iliac fossa pain without fever or vomiting	C-reactive protein (0.71 mg/dL)	Histopathology	No neoplasm
Ergenç, and Uprak (January 2016–January 2022, Turkey), retrospective study [20]	10 (8/2)	34.4 ± 17.2	All patients had right lower quadrant abdominal pain	A mean white-blood-cell count (WBC) of 13.5 × 10^9^/L (±5.02) and a mean neutrophil percentage of 70.6 (±7.82) (leukocytosis was observed in 7 patients and neutrophilia in 5 patients)	Histopathology (*n* = 10)	Sessile serrated adenoma (*n* = 1) and low-grade appendiceal mucinous neoplasm (*n* = 1)
Elkhawaga et al. (2022, Austria), case report [21]	1 (1/0)	71	1-day history of right iliac fossa pain	Right iliac fossa tenderness and rebound tenderness	Histopathology	NA
Abdulmomen et al. (2021, Saudi Arabia), case report [22]	1 (1/0)	35	1-day history of right lower quadrant abdominal pain that radiated to the left lower quadrant	Leukocytosis (11 × 10^9^/L) and CT scan revealed acute perforated appendicitis with early mass formation	Histopathology	NA
Wiliams et al. (2021, USA), case report [11]	1 (1/0)	85	Right lower quadrant abdominal pain	Mildly protuberant abdomen without significant guarding or rebound	CT	NA
Onafowokan et al. (2021, USA), case report [23]	1 (0/1)	23	Acute onset of severe right lower quadrant abdominal pain, nausea	Tenderness in the right lower quadrant and a positive Murphy’s sign	Histopathology	NA
Bujold-Pitre et al. (2021, Canada), case report [24]	1 (1/0)	72	2-day history of abdominal pain (left upper quadrant)	Leukocytosis (13.0 × 10^9^/L)	Histopathology	No neoplasm
Fiordaliso et al. (2020, Italy), case report [14]	1 (1/0)	68	5-day history abdominal pain. The pain was progressive in severity and was associated with constipation and fever.	Bilateral lower quadrant tenderness, leukocytosis (14 × 10^9^/L), and fever (38 °C)	Histopathology	NA
Memon et al. (2020, USA), case report [25]	1 (0/1)	52	1-day history of generalized abdominal pain, vomiting (once)	Mild diffuse tenderness to palpation with most pronounced pain in the right upper quadrant	Histopathology	Sessile serrated adenoma
Albeeshi et al. (2019, Saudi Arabia), case report [26]	1 (0/1)	28	2-day history of periumbilical pain, shifting to the right lower quadrant, associated with nausea and anorexia. No fever.	Tenderness at the right lower quadrant with positive rebound tenderness	Histopathology	NA
Hwala et al. (2019, Lebanon), case report [27]	1 (1/0)	44	2-day history of periumbilical pain (intermittent, without radiation), without nausea, vomiting or fever	Positive McBurney’s sign, leukocytosis (11.4 × 10^9^/L), neutrophils of 85.2%, and lymphocytes of 9%	Histopathology	NA
Vass et al. (2018, China), case report [28]	1 (0/1)	65	2-day history of intermittent pain in the right iliac fossa	NA	Histopathology	NA
Singh-Ranger and Mangalika (2018, United Kingdom), case report [29]	1 (1/0)	54	3-day history of cramping peri-umbilical abdominal pain that radiated to the right iliac fossa, nausea	Guarding and rebound in the right iliac fossa, leukocyte count (12 × 10^9^/L, neutrophil count (10 × 10^9^/L), and CRP (325 mg/dL)	Histopathology	NA
Ogawa et al. (2018, Japan), case report [30]	1 (1/0)	63	Hematochezia 3 h prior to admission	Rectal examination showed bloody stools, colonoscopy showed continuous bleeding from the orifice of the appendix	Histopathology	NA
Altieri et al. (2017, Italy), case report [12]	1 (1/0)	40	Right lower quadrant pain, vomiting, and fever	Abdominal tenderness and leukocytosis (11.93 × 10^9^/L)	Histopathology	NA
Constantin et al. (2017, Romania), case report [1]	1 (0/1)	50	Right lower quadrant abdominal pain (intermittent)	Guarding in the right iliac fossa and a positive psoas sign	Intra-operative	No neoplasm
Lourenço et al. (2011, Brazil), case report [31]	1 (1/0)	61	Constipation and tenderness in the lower right abdominal quadrant	Fever (38.4 °C) and leukocytosis (15.7 × 10^9^/L)	Histopathology	No neoplasm
Fernández Gómez-Cruzado et al. (2017, Spain), case report [32]	1 (1/0)	61	4-day history of lower quadrant pain	Leukocytosis (9.7 × 10^9^/L) and elevated CRP levels (CRP = 1.57 mg/dL)	CT	Carcinoid tumor
El-Saady (2016, Egypt), case report [33]	1 (1/0)	32	2-day history of diffuse periumbilical pain that shifted to the right iliac fossa and suprapubic areas within 6 h from onset, vomiting (once), constipation, and fever	Tender Mc-Burney point with rebound tenderness in the right iliac fossa, fever (38.1 °C), and leukocytosis (9 × 10^9^/L)	Histopathology	NA
Lange et al. (2015, Germany), case report [34]	1 (1/0)	52	4-day history of the right lower quadrant pain	CRP was slightly elevated (3.5 mg/dL)	Histopathology	No neoplasm
Subramanian et al. (2015, Singapore), case report [35]	1 (1/0)	50	7-day history of intermittent moderate pain in the right lower quadrant	Mild localized tenderness in the right lower quadrant	CT	NA
Yardimci et al. (January 2010–July 2015, Japan), retrospective study [36]	24 (17/7)	42.0 ± 11.4	NA	NA	Histopathology (*n* = 24)	None of the patients had an associated neoplasm
Zubieta- O’Farrill et al. (2014, Mexico), case report [13]	1 (0/1)	73	Right lower quadrant pain lasting three days, occurring five times over a four-month period prior to admission	Complete blood work was within normal range	Histopathology	No neoplasm
Patil et al. (2014, USA), case report [37]	1 (0/1)	61	Intermittent abdominal pain in the right lower quadrant, nausea, and anorexia	Afebrile and had a normal white-blood-cell count	CT	No neoplasm
Sohn et al. (January 2009–May 2011, South Korea), retrospective study [38]	38 (19/19)	49.0 ± 15.2	Right lower quadrant abdominal pain (*n* = 34, 89.5%), fever (*n* = 12, 31.5%), nausea (*n* = 18, 47.4%), vomiting (*n* = 8, 21.1%), and diarrhea (*n* = 4, 10.5%)	Tenderness, rebound tenderness, and muscle guarding were found (*n* = 16, 42.1%)	Histopathology (*n* = 38)	NA
Heffernan et al. (2009, USA) [39]	1 (0/1)	46	10-day history of right sided abdominal pain	Right lower quadrant tenderness, fever (38.7 °C), and leukocytosis (16 × 10^9^/L)	Histopathology	No neoplasm
Yamana et al. (January 2005–June 2008, Japan), retrospective study [40]	12 (10/2)	42.7 ± 15.4	NA	The mean leukocyte count at the time of the admission was 11,332 ± 4658 µ/L (8 patients had leukocytosis), the mean CRP level was 8.7 ± 8.9 mg/dL (6 patients had elevated CRP levels), and perforation was observed in 4 patients	Histopathology (*n* = 12)	None of the patients had an associated neoplasm
Käser et al. (June 1998–June 2008, Switzerland), retrospective study [41]	9 (NA)	33.3 ± 18.3	NA	4 patients had leukocytosis and 8 patients had elevated CRP levels	Histopathology (*n* = 9)	Neuroendocrine carcinoid (*n* = 2)
Kubota et al. (2006, Japan) [42]	1 (0/1)	30	4-day history of abdominal pain in right lower quadrant, fever, and mild anorexia	Localized tenderness in the right lower quadrant of abdomen, fever (37.4 °C), and leukocytosis (count of 11.0 × 10^9^/L)	Abdominal ultrasound sonography	No neoplasm
Lanthaler et al. (2004, Austria), case report [43]	1 (1/0)	39	10-day history of right lower-quadrant abdominal pain	Abdominal examination revealed right lower-quadrant pain with localized guarding, leukocytosis (18,000 g/dL), and elevated CRP levels (CRP = 8.9 mg/dL)	Histopathology	No neoplasm
Iki et al. (2001, Japan), case report [44]	1 (1/0)	84	4-day history of right lower quadrant abdominal pain and fever	Tenderness in the right lower quadrant region with peritoneal signs	Abdominal ultrasound sonography	NA

NA, not applicable; CT, compuer tomography; CRP, C-reactive protein; USA, the United States of America.

**Table 4:** Clinical presentation of appendiceal diverticulitis.

Symptoms	Characteristics	Onset
Abdominal pain in the right lower quadrant	Intermittent, insidious, and originates in right lower quadrant	2–13 days
Fever	Often absent	Variable
Anorexia, nausea, and emesis	Temperature of 38.4 °C or more	Variable

**Table 5:** Management, intraoperative and postoperative complications, length of hospital stay, and mortality in patients with appendiceal diverticulitis.

Author (Study Period, Country), Study Design	Management	Intraoperative Complications	Postoperative Complications	Length of Hospital Stay (Days)	Mortality
Abdelrahim et al. (2024, United Kingdom), case report [16]	Laparoscopic appendectomy	None	None	NA	0
Cadena et al. (2023, Colombia), case report [17]	Laparoscopic appendectomy	None	None	2	0
Laamiri et al. (2023, Tunisia), case report [18]	Laparoscopic appendectomy	None	None	2	0
Bonomo et al. (2022, Italy), case report [19]	Laparoscopic appendectomy	None	None	3	0
Ergenç, and Uprak (January 2016–January 2022, Turkey), retrospective study [20]	Open appendectomy (*n* = 8), appendectomy-midline laparotomy (*n* = 1), and laparoscopic appendectomy (*n* = 1)	None	None	2 (2–12)	0
Elkhawaga et al. (2022, Austria), case report [21]	Laparoscopic appendectomy	None	None	2	0
Abdulmomen et al. (2021, Saudi Arabia), case report [22]	Laparoscopic appendectomy	None	None	4	0
Wiliams et al. (2021, USA), case report [11]	Nonoperatively (watchful waiting)	NA	NA	NA	NA
Onafowokan et al. (2021, USA), case report [23]	Laparoscopic appendectomy	None	None	2	0
Bujold-Pitre et al. (2021, Canada), case report [24]	Laparoscopic right hemicolectomy	None	None	NA	0
Fiordaliso et al. (2020, Italy), case report [14]	Laparoscopic appendectomy	None	None	6	0
Memon et al. (2020, USA), case report [25]	Laparoscopic appendectomy	None	None	NA	0
Albeeshi et al. (2019, Saudi Arabia), case report [26]	Laparoscopic appendectomy	None	None	NA	0
Hwala et al. (2019, Lebanon), case report [27]	Laparoscopic appendectomy	None	None	NA	0
Vass et al. (2018, China), case report [28]	Laparoscopic appendectomy	None	None	NA	0
Singh-Ranger and Mangalika (2018, United Kingdom), case report [29]	Laparoscopic appendectomy	None	None	4	0
Ogawa et al. (2018, Japan), case report [30]	Laparoscopic appendectomy	None	None	4	0
Altieri et al. (2017, Italy), case report [12]	Appendectomy unspecified	None	None	4	0
Constantin et al. (2017, Romania), case report [1]	Open appendectomy	None	None	NA	0
Fernández Gómez-Cruzado et al. (2017, Spain), case report [32]	Open appendectomy	None	None	NA	0
El-Saady (2016, Egypt), case report [33]	Open appendectomy	None	None	NA	0
Lange et al. (2015, Germany), case report [34]	Laparoscopic appendectomy	None	None	4	0
Subramanian et al. (2015, Singapore), case report [35]	Laparoscopic appendectomy	None	None	3	0
Yardimci et al. (January 2010–July 2015, Japan), retrospective study [36]	NA	NA	NA	NA	NA
Zubieta-O’Farrill et al. (2014, Mexico), case report [13]	Laparoscopic appendectomy	None	None	2	0
Patil et al. (2014, USA), case report [37]	Laparoscopic appendectomy	None	None	3	0
Sohn et al. (January 2009–May 2011, South Korea), retrospective study [38]	Appendectomy, unspecified	NA	NA	6.8 ± 3.4	NA
Lourenço et al. (2011, Brazil), case report [31]	Open right hemicolectomy and appendectomy	None	None	NA	0
Heffernan et al. (2009, USA) [39]	Open appendectomy	None	None	NA	0
Yamana et al. (January 2005– June 2008, Japan), retrospective study [40]	Appendectomy, unspecified	NA	NA	8.7 ± 4.86	NA
Käser et al. (June 1998–June 2008, Switzerland), retrospective study [41]	Appendectomy, unspecified	NA	NA	NA	NA
Kubota et al. (2006, Japan) [42]	Appendectomy, unspecified	None	None	3	0
Lanthaler et al. (2004, Austria), case report [43]	Appendectomy, unspecified	None	None	2	0
Iki et al. (2001, Japan), case report [44]	Appendectomy, unspecified	None	None	NA	0

NA, not applicable; USA, the United States of America.

**Table 6:** Key distinguishing features between appendiceal diverticulitis and acute appendicitis.

Feature	Appendiceal Diverticulitis	Acute Appendicitis
Onset of symptoms	Insidious, intermittent, 2–14 days	Sudden, progressive within 24–48 h
Age group	More common > 30 years	More common < 30 years
Pain localization	Initially diffuse, then localizing	Early periumbilical, then RLQ pain
Leukocytosis	Often > 15 × 10^9^/L	Often < 15 × 10^9^/L
CRP levels	Frequently elevated (>20 mg/L)	May be mildly elevated or normal
Perforation rate	30–70%	10–20%
Neoplasm association	Present in up to 20% of cases	Rare
Imaging findings (CT/US)	Appendiceal diverticula, wall thickening, peridiverticulitis	Enlarged, non-compressible appendix with periappendiceal fat stranding
AIR score	Might be lower compared to acute appendicitis AIR score	Might be higher compared to appendiceal diverticulitis AIR score

RLQ, right lower quadrant; CRP, C-reactive protein; CT, computed tomography; US, abdominal ultrasound; AIR score, the Appendicitis Inflammatory Response score.

## Data Availability

The data are not publicly available since they contain information that could compromise the privacy of the research participant.
